# African swine fever virus pS273R antagonizes stress granule formation by cleaving the nucleating protein G3BP1 to facilitate viral replication

**DOI:** 10.1016/j.jbc.2023.104844

**Published:** 2023-05-19

**Authors:** Tingting Li, Xuewen Li, Xiao Wang, Xin Chen, Gaihong Zhao, Chuanxia Liu, Miaofei Bao, Jie Song, Jiangnan Li, Li Huang, Jun Rong, Kegong Tian, Junhua Deng, Jianzhong Zhu, Xuehui Cai, Zhigao Bu, Jun Zheng, Changjiang Weng

**Affiliations:** 1 Division of Fundamental Immunology, State Key Laboratory for Animal Disease Control and Prevention, Harbin Veterinary Research Institute of Chinese Academy of Agricultural Sciences, Harbin, China; 2 Heilongjiang Provincial Key Laboratory of Veterinary Immunology, Harbin, China; 3 College of Life Sciences, Yangtze University, Jingzhou, China; 4 National Research Center for Veterinary Medicine, Luoyang, China; 5 Luoyang Putai Biotechnology Co, Ltd, Luoyang, China; 6 College of Veterinary Medicine, Yangzhou University, Yangzhou, China

**Keywords:** African swine fever virus, G3BP1, stress granules, IFN production, pS273R, viral replication

## Abstract

Cytoplasmic stress granules (SGs) are generally triggered by stress-induced translation arrest for storing mRNAs. Recently, it has been shown that SGs are regulated by different stimulators including viral infection, which is involved in the antiviral activity of host cells to limit viral propagation. To survive, several viruses have been reported to execute various strategies, such as modulating SG formation, to create optimal surroundings for viral replication. African swine fever virus (ASFV) is one of the most notorious pathogens in the global pig industry. However, the interplay between ASFV infection and SG formation remains largely unknown. In this study, we found that ASFV infection inhibited SG formation. Through SG inhibitory screening, we found that several ASFV-encoded proteins are involved in inhibition of SG formation. Among them, an ASFV S273R protein (pS273R), the only cysteine protease encoded by the ASFV genome, significantly affected SG formation. ASFV pS273R interacted with G3BP1 (Ras-GTPase-activating protein [SH3 domain] binding protein 1), a vital nucleating protein of SG formation. Furthermore, we found that ASFV pS273R cleaved G3BP1 at the G140–F141 to produce two fragments (G3BP1-N_1–140_ and G3BP1-C_141–456_). Interestingly, both the pS273R-cleaved fragments of G3BP1 lost the ability to induce SG formation and antiviral activity. Taken together, our finding reveals that the proteolytic cleavage of G3BP1 by ASFV pS273R is a novel mechanism by which ASFV counteracts host stress and innate antiviral responses.

African swine fever (ASF), caused by the ASF virus (ASFV), is a highly contagious and fatal disease that causes high mortality in domestic pigs and wild boar (1). In recent years, ASF has rapidly spread to Eastern European countries, Timor-Leste of Oceania, and many Asian countries (2). So far, there are no commercial vaccines and effective antiviral drugs available to prevent and control ASFV infections. Thereby, ASFV poses a potential threat to the pig industry in the world (3).

ASFV is the only member of the family Asfarviridae, genus *Asfivirus*, which contains a linear double-stranded DNA genome of about 170 to 194 kbp in length and encodes more than 150 proteins (4). Among these viral proteins, pS273R encoded by ASFV at the late stages of viral infection is the only known SUMO-1 cysteine protease, which catalyzes the maturation of the pp220 and pp62 precursors *via* proteolytical cleavage for ASFV assembly (5, 6). In addition, pS273R could regulate host immune responses to promote viral replication (7, 8, 9). For example, pS273R decreased the expression of FoxJ1, which is a host antiviral factor (7). Our previous study also demonstrated that pS273R inhibited pyroptosis by noncanonical cleavage of gasdermin D to promote ASFV replication (8). These reports suggest that pS273R plays pleiotropic roles during ASFV infection.

Stress granules (SGs) are nonmembranous RNA–protein complexes composed of translationally stalled mRNAs, 40S ribosomes, eukaryotic initiation factors, and various RNA-binding proteins (*e.g.*, Ras-GTPase-activating protein [SH3 domain] binding protein 1 [G3BP1], G3BP2, TIA1-related protein [TIAR], protein kinases (protein kinase R [PKR]), cell cycle–associated protein 1 (CAPRIN1), ubiquitin binding protein 2-like [UBAP2L]), which are formed in the cytoplasm in response to different cellular stresses, including virus infection (10). When stresses occur, RNA-binding proteins serve as nodes to bind dispersed RNAs, which forms a protein–RNA interaction network (11). And then the network could drive liquid–liquid phase separation, finally resulting in SG assembly. Among these RNA-binding proteins, G3BP1 is a central node of this network and functions as a molecular switch for SG assembly (12), where G3BP1 triggering SG formation depends on its intact structure (12).

Currently, the functional role of these SGs remains controversial. However, accumulating evidence points to the role of these SGs as an immune signaling platform in antiviral defense (13, 14, 15). For instance, during the Sendai virus infection, SG formation is important for interferon (IFN)-β production and restriction of viral replication (15). Many viruses have evolved several strategies to inhibit the formation of SGs, suggesting that SGs serve an antiviral function. Among these key strategies, there is a direct approach that virus-encoded proteases interacted with and cleaved the critical components of SGs, such as G3BP1. For instance, at later stages of encephalomyocarditis virus infection, cleavage of G3BP1 by the encephalomyocarditis virus protease 3C resulted in SG dissolution concomitant with a reduced IFN-β and cytokine release, which could be rescued by expressing a cleavage-resistant G3BP1 mutant (14). This strategy also was observed during foot-and-mouth disease virus (16), feline calicivirus (17) or coxsackievirus type B3 infection (18). In addition, indirect approaches are adopted by several viral proteins to prevent PKR activation such as by indirect masking or degrading the dsRNA (*e.g.*, coronavirus, adenovirus, and so forth) (19, 20, 21) or to redirect SG factors to viral replication complexes (*e.g.*, Chikungunya virus, Sindbis virus, and so forth) (22, 23). However, whether ASFV has also evolved strategic mechanisms to modulate SG formation is still unknown.

In this study, we found that ASFV infection inhibited SG formation. Meanwhile, we analyzed the effect of 102 ASFV proteins on SG formation through an unbiased screening *in vitro* and found several proteins, including pS273R. Furthermore, we found that ASFV pS273R cleaved G3BP1, a vital SG-nucleating protein, at the G140–F141, which impaired SG formation and relieved the inhibitory effect of SGs on ASFV replication. Taken together, our finding reveals that proteolytic cleavage of G3BP1 by ASFV pS273R is a novel mechanism by which ASFV counteracts host stress and innate antiviral response.

## Results

### ASFV infection inhibits SG formation

**Figure 1:**
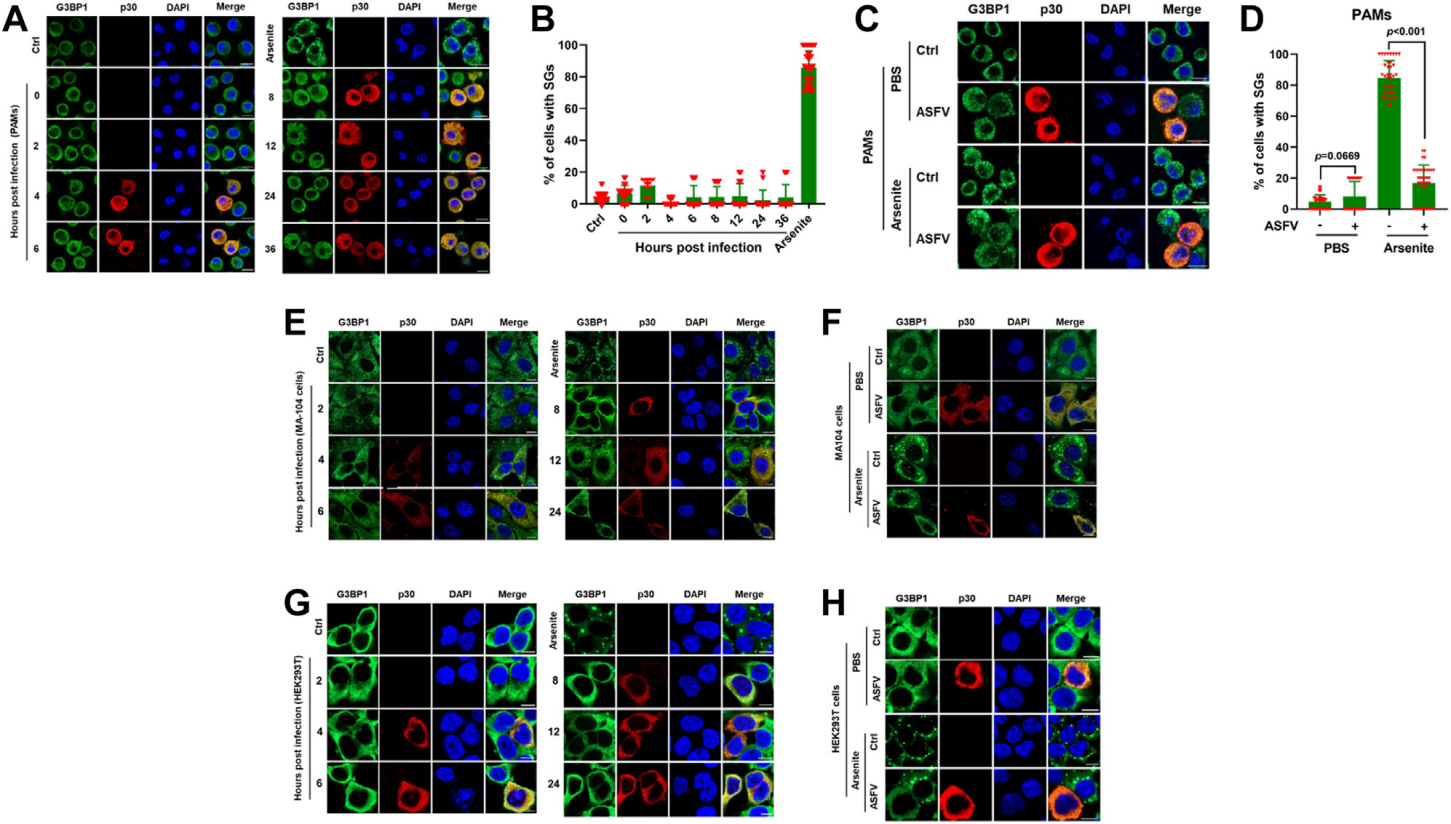
**ASFV infection inhibits the formation of SGs.***A*, PAMs were infected with ASFV (MOI of 1) and then fixed at indicated time points. The fixed cells were stained with either rabbit polyclonal-specific antibodies for G3BP1 (*green*) or mouse polyclonal-specific antibodies for p30 (*red*). The cells were treated with arsenite (0.5 μM) for 1 h as a positive control. Nuclei were stained with DAPI (*blue*). The cells were analyzed by confocal microscopy. Scale bar represents 10 μM. *B*, the percentage of SG-positive infected cells to the infected cells, which was calculated in 30 random fields, was presented as mean ± SD. *C*, PAMs were infected with ASFV (MOI of 1) for 24 h and then treated or untreated with arsenite for another 1 h before indirect immunofluorescence was performed. The cells were fixed and stained with either rabbit polyclonal-specific antibodies for G3BP1 (*green*) or mouse polyclonal-specific antibodies for p30 (*red*). Nuclei were stained with DAPI (*blue*). Scale bar represents 10 μM. *D*, the percentage of SG-positive cells to the infected cells, which was calculated in 30 random fields, was presented as mean ± SD. *p* Values were calculated with an unpaired *t* test. *E*, MA-104 cells were infected with ASFV (MOI of 2) as described in *A*. Scale bar represents 10 μM. *F*, MA-104 cells were infected with ASFV (MOI of 2) as described in *C*. Scale bar represents 10 μM. *G*, HEK293T cells were infected with ASFV (MOI of 2) as described in *A*. Scale bar represents 10 μM. *H*, HEK293T cells were infected with ASFV (MOI of 2) as described in *C*. Scale bar represents 10 μM. ASFV, African swine fever virus; DAPI, 4′,6-diamidino-2-phenylindole; G3BP1, Ras-GTPase-activating protein (SH3 domain) binding protein 1; HEK293T, human embryonic kidney 293T cell line; MOI, multiplicity of infection; PAM, porcine alveolar macrophage; SG, stress granule.

To investigate the relationship between ASFV and SG formation, we performed the immunofluorescence assay to monitor the dynamics of SGs during ASFV infection. Porcine alveolar macrophages (PAMs), the natural targeted cells of ASFV (4), were mock infected or infected with ASFV at a multiplicity of infection (MOI) of 1 or treated with arsenite (0.5 μM, 1 h), a common reagent used to induce SGs *via* oxidative stress, as a positive control. At indicated time points, SGs were identified by staining for G3BP1 and TIAR, which are the marker proteins of SGs (24). And ASFV-infected PAMs were identified with antisera specific for ASFV p30, a viral early protein. We found that arsenite robustly induced SG formation, whereas no obvious SGs were observed in ASFV-infected PAMs, indicating ASFV infection might inhibit SG formation (Figs. 1, *A* and *B* and S1, *A* and *B*). To confirm the hypothesis, we detected whether ASFV infection inhibited arsenite-induced SG formation. We observed no obvious SG formation in ASFV-infected PAMs following treatment with arsenite (Fig. 1*C*). And among these ASFV-infected PAMs with arsenite treatment, the percentage of the cells displaying G3BP1-positive SGs was significantly lower than that in cells treated with arsenite alone (Fig. 1*D*). Similar results were obtained using ASFV-infected MA-104 cells, a suitable cell line for ASFV growth (25) (Fig. 1, *E* and *F*). Recently, human embryonic kidney 293T (HEK293T)-adapted viruses have been obtained (26); therefore, we detected whether ASFV infection can inhibit SG formation in HEK293T cells. We found that SG formation was also inhibited in ASFV-infected HEK293T cells (Fig. 1, *G* and *H*). Taken together, these data suggest that ASFV infection inhibits SG formation.

### Screening of the ASFV-encoded proteins that inhibit SG formation *in vitro*

**Figure 2:**
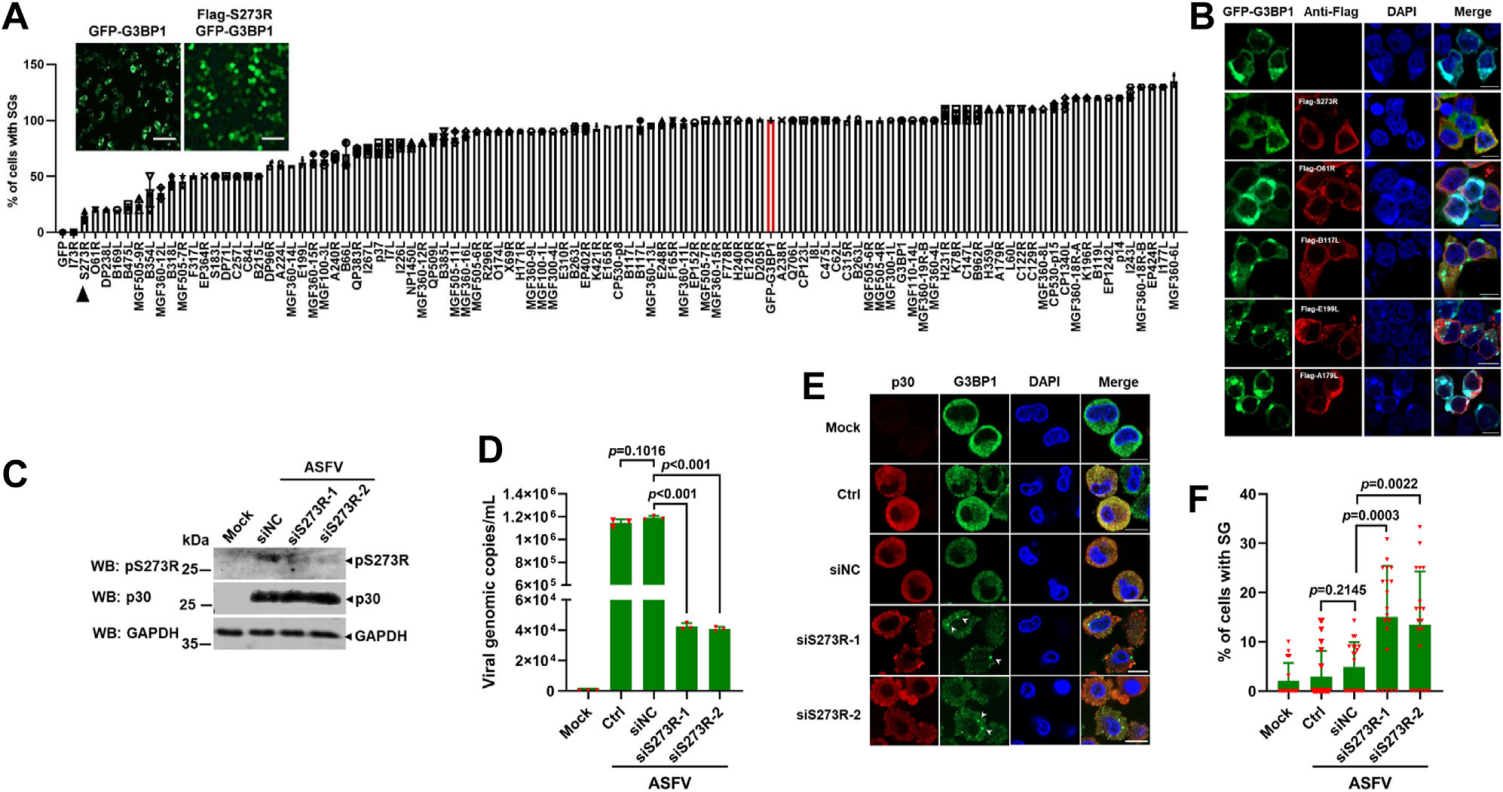
**Screening of the ASFV-encoded proteins that inhibited SG formation *in vitro.****A*, HEK293T cells were cotransfected with plasmids expressing GFP-G3BP1 and the indicated plasmids encoding ASFV proteins, respectively. At 24 h, the cells were fixed and stained with mouse-specific antibodies for FLAG tag (*red*). Nuclei were stained with DAPI (*blue*), then monitored, and counted the percentage of cells forming SGs in 20 random fields by immunofluorescence microscopy. Scale bar represents 10 μM. *B*, the panel showed part representative images of the inhibition of SG formation by ASFV-encoded proteins. Scale bar represents 10 μM. *C*–*E*, PAMs were transfected individually with two different siRNAs targeting the S273R gene and then infected with ASFV at an MOI of 1. At 24 hpi, the expression of pS273R, p30, and GAPDH was detected by Western blotting (*C*), and the genomic DNA copy number of ASFV (*D*) and SG formation in ASFV-infected PAMs were observed and analyzed by confocal microscopy as described for Figure 1*A*. *E*, scale bar represents 10 μM. *F*, the percentage of SG-positive cells to the infected cells, which was calculated in 20 random fields in (*E*), was presented as mean ± SD. *p* Values were calculated with an unpaired *t* test. ASFV, African swine fever virus; DAPI, 4′,6-diamidino-2-phenylindole; G3BP1, Ras-GTPase-activating protein (SH3 domain) binding protein 1; HEK293T, human embryonic kidney 293T cell line; hpi, hours postinfection; MOI, multiplicity of infection; SG, stress granule.

Increasing evidence showed that various viruses-encoded proteins could regulate the process of SG formation (10, 27). To identify which ASFV protein could inhibit SG formation, each of the 102 ASFV proteins is coexpressed separately with GFP-G3BP1, which can induce SG formation without any additional stresses (28). The percentage of cells displaying G3BP1-induced SGs was evaluated *via* immunofluorescence microscopy. As shown in Figure 2, *A* and *B*, several viral proteins could significantly inhibit G3BP1-induced SGs, including pI73R, pS273R, and pO61R. In this study, we focused on pS273R because pS273R had the stronger effect on inhibiting SG formation, and our previous data showed that pS273R was predicted to be associated with G3BP1 by pull down and mass spectrometry with His-S273R proteins (8). To test the effect of pS273R on SG formation during ASFV infection, we used the siRNA to decrease the pS273R expression during viral infection (Fig. 2*C*). We found that knockdown of pS273R expression reduced the virions in infected PAMs (Fig. 2*D*), whereas SG formation was observed in ASFV-infected PAMs (Fig. 2, *E* and *F*). So ASFV pS273R was chosen for subsequent studies on the mechanism of inhibiting SG formation.

### ASFV pS273R protease interacts with and cleaves G3BP1

**Figure 3:**
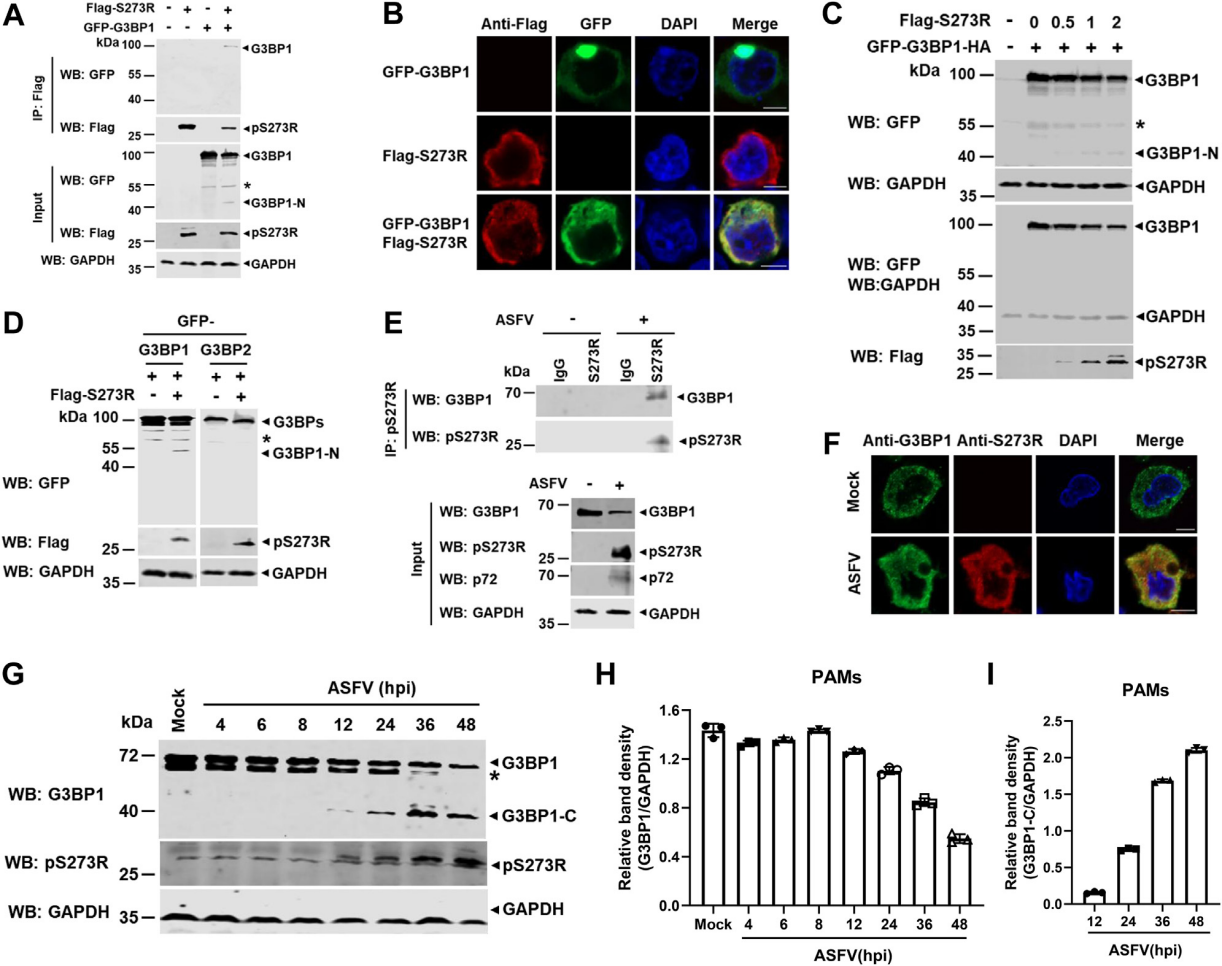
**ASFV pS273R interacts with and cleaves G3BP1 *in vitro*.***A*, HEK293T cells were transfected with a plasmid encoding G3BP1 containing a GFP tag located at its N terminus (GFP-G3BP1), in combination with a plasmid expressing FLAG-tagged pS273R or empty vector as indicated. The cell lysates were coimmunoprecipitated with an anti-FLAG antibody. The immunoprecipitants and aliquots of the whole-cell lysates were subjected to Western blot analysis with anti-GFP, anti-FLAG, or anti-GAPDH antibodies. *B*, HEK293T cells were transfected with a plasmid expressing FLAG-S273R or GFP-tagged G3BP1 alone or both. The cells were probed with mouse anti-FLAG monoclonal antibody (*red*). Green fluorescence indicates GFP-G3BP1. Blue florescence indicates cell nuclei stained with DAPI. Scale bar represents 5 μM. *C*, HEK293T cells were transfected with a plasmid expressing GFP-G3BP1-HA alone or together with increasing doses (0, 0.5, 1, or 2 μg) of a plasmid encoding FLAG-pS273R. The cell lysates were analyzed by Western blot with the indicated antibodies under long or short exposure conditions. *D*, HEK293T cells were transfected with a plasmid expressing GFP-tagged G3BP1 or G3BP2 along with a plasmid encoding FLAG-S273R. At 36 hpt, the cell lysates were analyzed by Western blot with the indicated antibodies. *E*, PAMs were infected with ASFV at an MOI of 1 for 36 h, and then the cell lysates were coimmunoprecipitated with mouse IgG or anti-pS273R polyclonal antibody (pAb). The cell lysates and immunoprecipitants were analyzed by Western blotting with anti-G3BP1 and anti-pS273R pAbs. *F*, PAMs were infected with ASFV at an MOI of 1 for 36 h. The cells were probed with rabbit anti-G3BP1 monoclonal antibody (mAb) (*green*) and mouse anti-pS273R mAb (*red*). The cell nuclei (*blue*) were stained with DAPI. Scale bar represents 5 μM. *G*, PAMs were noninfected or infected with ASFV at MOIs of 2. At the indicated time points, the cell lysates were analyzed by Western blot with antibodies against G3BP1, pS273R, and GAPDH. ∗ indicated the bands that may be unknown modified G3BP1. *H* and *I*, quantitation of G3BP1/GAPDH ratio (*H*) or G3BP1-C/GAPDH ratio (*I*) from ImageJ analysis in (*G*). ASFV, African swine fever virus; DAPI, 4′,6-diamidino-2-phenylindole; G3BP1, Ras-GTPase-activating protein (SH3 domain) binding protein 1; HA, hemagglutinin; HEK293T, human embryonic kidney 293T cell line; IgG, immunoglobulin G; MOI, multiplicity of infection; PAM, porcine alveolar macrophage.

To further confirm the interaction between pS273R and G3BP1, FLAG-pS273R or GFP-G3BP1 alone or both were coexpressed in HEK293T cells, and the interaction and the subcellular colocalization of the two proteins were examined. As shown in Figure 3, *A* and *B*, pS273R interacted with and colocalized with G3BP1 in the cytoplasm. In addition, when FLAG-pS273R and GFP-G3BP1 were ectopically coexpressed, the two specific bands, the GFP-G3BP1 (approximately100 kDa) and its N-terminal region (between 40 and 55 kDa), could be immunoblotted with GFP antibody (Fig. 3*A*). The result promotes us to wonder whether ASFV pS273R may cleave swine G3BP1 *in vitro*.

To further test whether G3BP1 is cleaved by ASFV pS273R, we ectopically cotransfected with a plasmid expressing GFP-G3BP1-hemagglutinin (HA) and increasing amounts of a plasmid encoding FLAG-S273R in HEK293T cells. As shown in Figure 3*C*, the intensity of an N-terminal fragment of G3BP1 (designated G3BP1-N) increased along with the increased protein level of FLAG-S273R, whereas the intensity of G3BP1 decreased along with the increased protein level of FLAG-S273R. These results suggested that G3BP1 was cleaved by ASFV pS273R in a dose-dependent manner. In addition, we also analyzed the impact of pS273R on G3BP2, a G3BP1 homolog protein recruited to SGs (29) and found that pS273R had no impact on G3BP2 integrity (Fig. 3*D*).

To test whether ASFV pS273R interacts with endogenous G3BP1 in ASFV-infected PAMs, PAMs were isolated and infected with ASFV HLJ/18 and subjected to a coimmunoprecipitation (co-IP) assay. The interaction of pS273R with endogenous G3BP1 was observed (Fig. 3*E*). Meanwhile, we also noticed that pS273R colocalized with endogenous G3BP1 in the cytoplasm of PAMs during ASFV infection (Fig. 3*F*). These data supported that ASFV pS273R specifically interacted with G3BP1. Next, we analyzed the integrity of G3BP1 during ASFV infection. PAMs were infected with different doses (1 or 2 MOI) of ASFV for the indicated time points. And then, the whole-cell lysates were subjected to Western blotting using a G3BP1 polyclonal antibody that could recognize epitopes in the C terminus (amino acids [aa] 167–465) of G3BP1 (Figs. 3*G* and S4). Subsequently, we tested the integrity of G3BP1 during ASFV infection and found that an approximately 40 kDa cleavage product (designated G3BP1-C) could be detected after pS273R was expressed from 8 hours post infection (hpi) onward (Fig. 3*G*). And the amount of the full-length G3BP1 gradually decreased, whereas the amount of cleaved product (G3BP1-C) increased from 8 hpi onward (Fig. 3, *H* and *I*). These data suggested that ASFV pS273R interacted with and cleaved G3BP1 *in vitro*.

### ASFV pS273R cleaves G3BP1 at Gly140, which depends on its enzymatic activity

**Figure 4:**
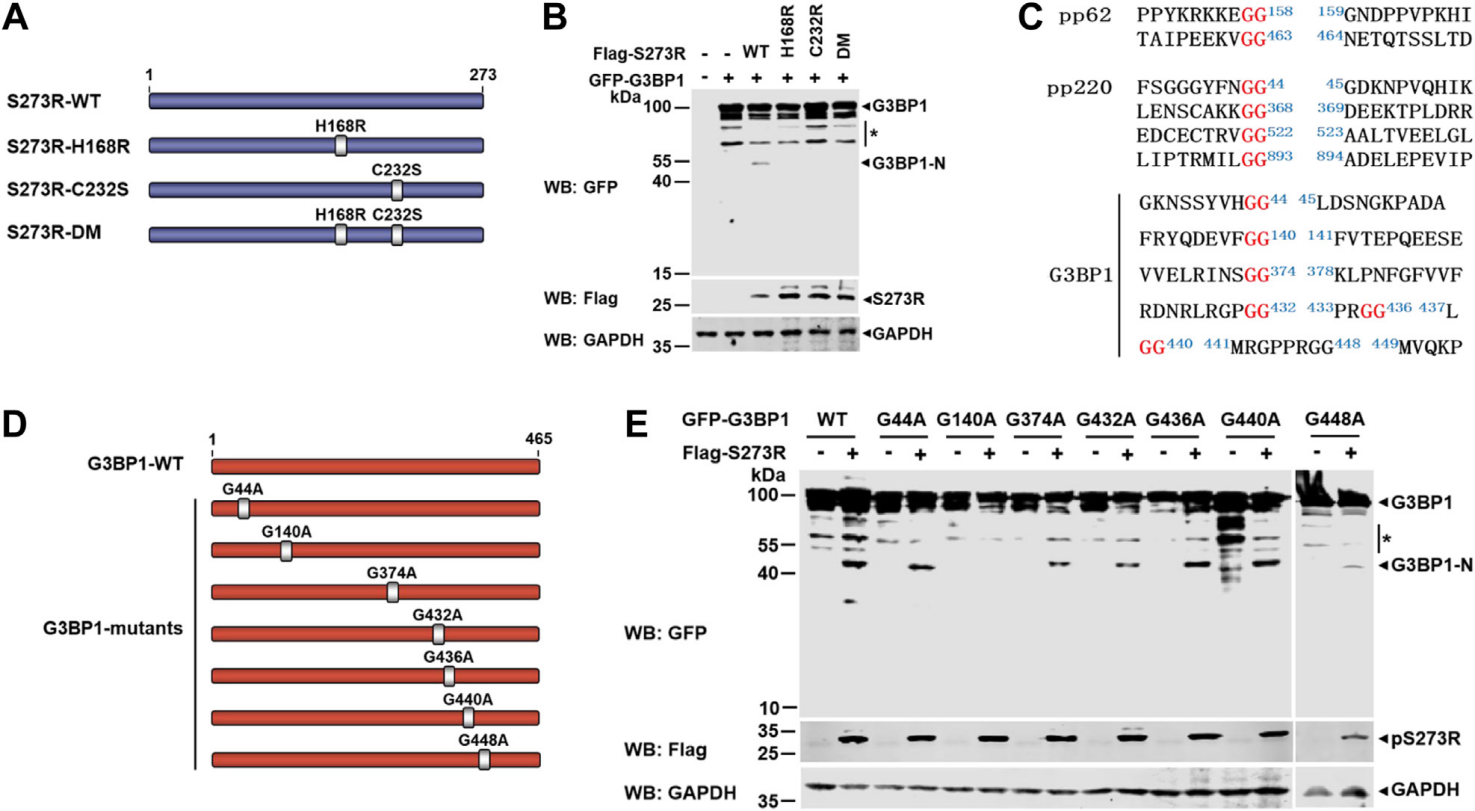
**ASFV pS273R cleaves G3BP1 at Gly140, which is depended on its enzymatic activity.***A*, schematic of ASFV pS273R constructs showing the positions of ASFV pS273R protease activity sites (labeled in *white*) at H168R and C232S. *B*, HEK293T cells were transfected with a plasmid encoding GFP-tagged G3BP1 alone or together with a plasmid expressing FLAG-pS273R or its mutants of inactive protease sites. The cell lysates were prepared and analyzed by Western blot. *C*, alignment of the ASFV S273R cleavage sites within pp62 and pp220 (*above*) and putative ASFV pS273R cleavage sites within swine G3BP1 (*bottom*). *D*, schematic of swine G3BP1 showing the position of the pS273R-cleaved putative sites. The glycine residues (G) at 44, 140, 374, 432, 436, 440, or 448 within swine G3BP1 were replaced with alanine residues (A). *E*, HEK293T cells were transfected with a plasmid expressing GFP-tagged G3BP1 or its mutants alone or in combination with a plasmid expressing FLAG-S273R. At 36 hpt, the cell lysates were analyzed by Western blot. ∗ indicated the bands that may be unknown modified G3BP1. ASFV, African swine fever virus; G3BP1, Ras-GTPase-activating protein (SH3 domain) binding protein 1; HEK293T, human embryonic kidney 293T cell line.

The pS273R is a specific SUMO-1 cysteine protein (30). It was reported that His168 and Cys232 were vital amino acids for the catalytic activity of pS273R (6). To test whether the protease activity of ASFV pS273R is required for G3BP1 cleavage, four previously constructed plasmids expressing pS273R-WT (WT and three catalytically inactive pS273R mutants (pS273R-HA168R, C232S, and H168R/C232S) were used (Fig. 4*A*). Subsequently, we compared the abilities of pS273R-WT and its three catalytically inactive forms to cleave the coexpressed GFP-G3BP1 (Fig. 4*B*). The result showed that only pS273R-WT induced G3BP1 cleavage rather than its mutants, indicating that G3BP1 is a proteolytic substrate for pS273R.

Based on previous reports, ASFV pS273R preferentially cleaves Gly-Gly (G-G) amino acid pairs within pp62 and pp220 (31). To search and confirm the potential pS273R cleavage sites in the sequence of G3BP1, we examined the amino acid sequence of swine G3BP1 for potential ASFV pS273R cleavage sites and found that seven regions bearing several glycines (G) resemble the signature G-G sequences of the proteolytic sites of ASFV pS273R (Fig. 4*C*, *bottom*). Therefore, we defined these putative cleavage sites. A series of G3BP1 mutants was constructed by the substitution of alanine acid for glycine acid (such as G44A, G140A, G374A, G432A, G436A, G440A, G448A) (Fig. 4*D*). G3BP1 or its mutants were ectopically coexpressed with FLAG-pS273R in HEK293T cells to identify the actual cleavage sites, respectively. As shown in Figure 4*E*, G3BP1-G140A substitution rendered G3BP1 resistant to ASFV pS273R cleavage, and other G3BP1 mutants did not prevent cleavage mediated by ASFV pS273R. These results suggested that pS273R mediated the cleavage of G3BP1 at G140–F141.

### The cleavage fragments of G3BP1 by pS273R are unable to induce SGs

**Figure 5:**
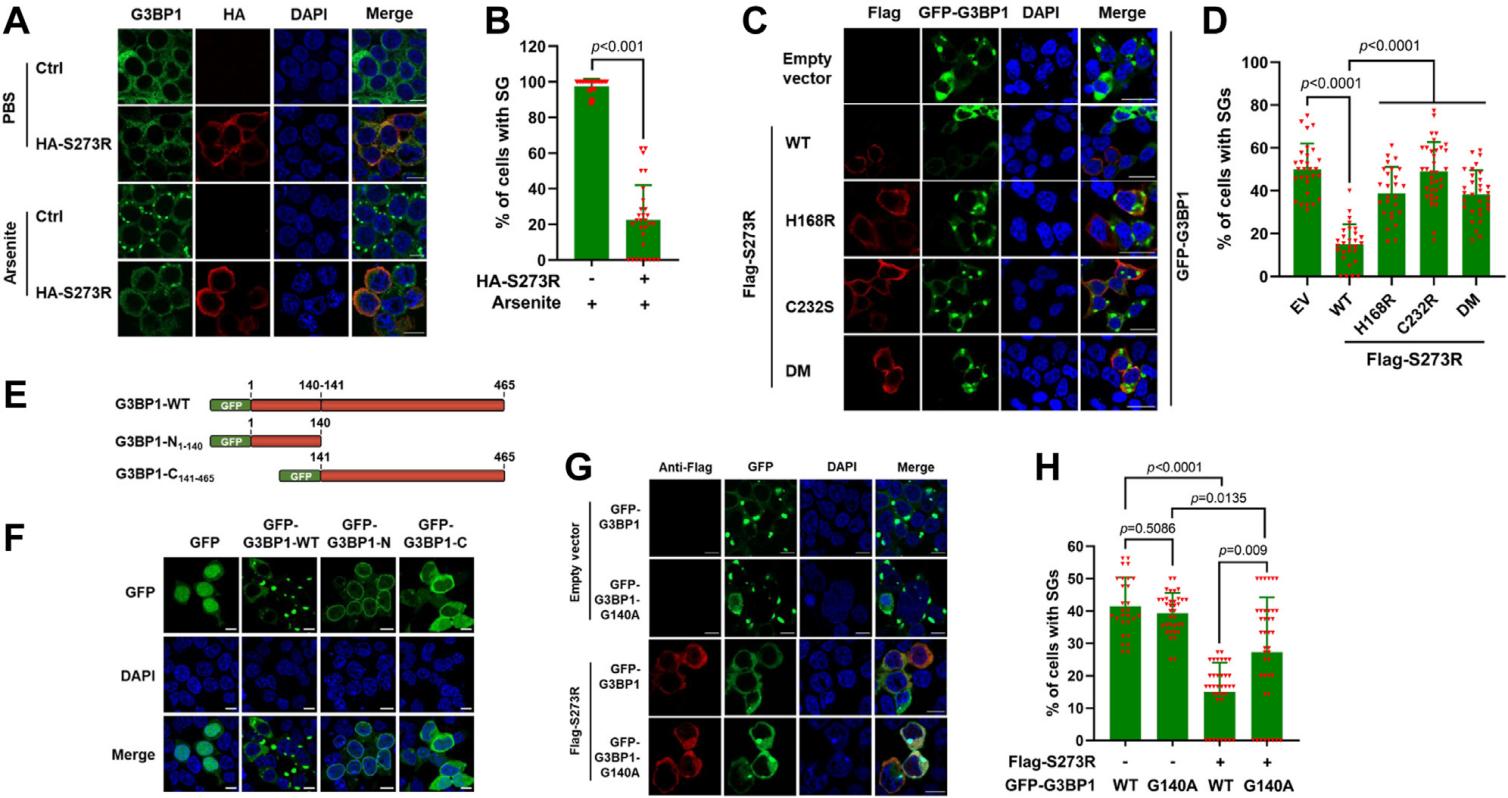
**The cleavage fragments of G3BP1 by pS273R are unable to induce SG assembly.***A*, HEK293T cells were transfected with a plasmid expressing HA-S273R or empty vector (EV) for 24 h and then treated or untreated with arsenite (0.5 μM) for another 30 min. Subsequently, the cells were fixed and immunostained with a G3BP1 antibody for G3BP1 (*green*) and a monoclonal HA antibody for HA-pS273R (*red*). Nuclei were stained with DAPI (*blue*). Scale bar represents 10 μM. *B*, the percentage of SG-positive cells, which was calculated in 30 random fields, presented as mean ± SD in (*A*). *p* Values were calculated with an unpaired *t* test. *C*, HEK293T cells were transfected with a GFP-G3BP1 expression plasmid respectively together with FLAG-S273R, its protease inactivity mutants, or empty vector (EV) expression plasmids, respectively. The cells were then fixed and stained with FLAG-tagged antibodies for FLAG-S273R and its mutants (*red*). Nuclei were stained with DAPI (*blue*). The cells were analyzed by confocal microscopy. Scale bar represents 10 μM. *D*, the percentage of cells containing SGs out of cells expressing GFP-G3BP1 alone or together with pS273R or its mutants was calculated in 30 random fields in (*C*). Data are represented as means ± SD. *p* Values were calculated with an unpaired *t* test. *E*, schematic diagrams of G3BP1 and its cleavage fragments produced by ASFV pS273R protease. *F*, HEK293T cells were transfected with a plasmid expressing GFP-tagged G3BP1, G3BP1-N_1–140_, and G3BP1-C_141–465_ for 24 h, fixed, and then SG formation was detected by confocal microscopy. Representative views of SG formation are shown in (*F*). Scale bar represents 10 μM. *G*, HEK293T cells were transfected with a GFP-G3BP1 or GFP-G3BP1-G140A expression plasmid together with a FLAG-S273R or empty vector (EV) expression plasmid. The cells were then fixed and stained with FLAG-tagged antibodies for FLAG-S273R (*red*). Nuclei were stained with DAPI (*blue*). SG formation was observed by confocal microscopy. Scale bar represents 10 μM. *H*, the percentage of cells containing SGs out of cells expressing GFP-G3BP1 alone or together with pS273R was calculated in 30 random fields in (*G*). Data are represented as means ± SD. *p* Values were calculated with an unpaired *t* test. DAPI, 4′,6-diamidino-2-phenylindole; G3BP1, Ras-GTPase-activating protein (SH3 domain) binding protein 1; HA, hemagglutinin; HEK293T, human embryonic kidney 293T cell line; SG, stress granule.

Previous reports showed that GFP-G3BP1 has been widely used as a reliable marker of SGs, and GFP-G3BP1 triggers SG formation depending on its intact structure (12, 28). Of note, we observed that there were no obvious SG formation in pS273R-expressed HEK293T cells following treatment with arsenite (0.5 μM, 30 min) that induced G3BP1-dependent SGs (32) (Fig. 5, *A* and *B*). Meanwhile, we found that pS273R inhibited SG formation induced by the overexpression of G3BP1, which is depended on pS273R enzymatic activity (Fig. 5, *C* and *D*). The aforementioned data raised the possibility that pS273R-cleaved G3BP1 might inhibit the formation of SGs. To test this hypothesis, we constructed two plasmids expressing swine G3BP1-N_1–140_ and G3BP1-C_141–465_ that mimic cleavage products generated by ASFV pS273R (Fig. 5*E*). And then, full-length G3BP1 and its cleaved fragments were ectopically expressed in HEK293T cells, and the SG formation was monitored by the immunofluorescence microscope. We found that overexpression of intact G3BP1 could induce SG formation as previous studies (28), whereas the G3BP1-N_1–140_ and G3BP1-C_141–465_ did not (Fig. 5*F*). To test whether the cleaved fragments of G3BP1 by pS273R have effect on G3BP1-mediated SG formation, GFP-G3BP1 was coexpressed with HA-G3BP1-N_1–140_ or HA-G3BP1-C_141–465_. The results showed that overexpression of HA-G3BP1-N_1–140_ or HA-G3BP1-C_141–465_ did not interfere with GFP-G3BP1-induced SG formation (Fig. S2, *A* and *B*). These results suggested that pS273R-mediated cleavage of G3BP1 suppresses SG formation.

To further confirm these results, we analyzed the impact of G140A mutation on pS273R-mediated inhibition of SG formation. GFP-G3BP1 or GFP-G3BP1-G140A was coexpressed with pS273R or empty vector (EV) in HEK293T cells. We found that coexpression of GFP-G3BP1 or GFP-G3BP1-G140A, together with an EV, resulted in obvious SG formation, and both had no difference (Fig. 5, *G* and *H*), suggesting that G140-replaced A of G3BP1 did not affect its ability to induce SGs. In contrast, a significant decrease in SG formation could be observed in cells coexpressing GFP-G3BP1 with pS273R, whereas GFP-G3BP1-G140A, a pS273R cleavage–resistant G3BP1 mutant, at least partially restored SG formation. These data indicate that pS273R-cleaved G3BP1 inhibits SG formation.

### The effect of G3BP1 and its cleaved fragments on ASFV replication

**Figure 6:**
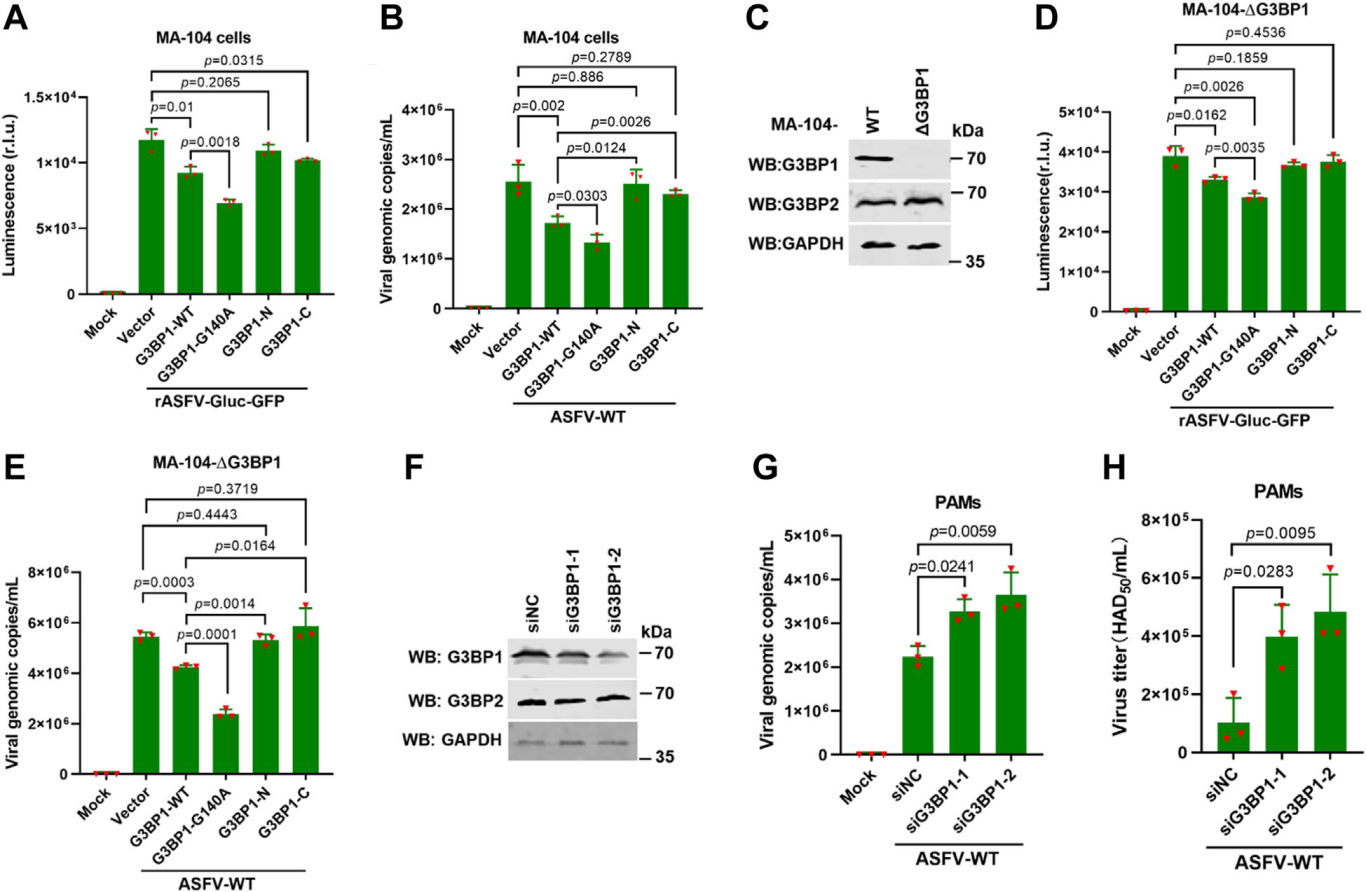
**The effect of G3BP1 and its cleaved fragments on ASFV replication.***A*, MA-104 cells were transfected with the designated GFP-G3BP1 expression plasmids or empty vector, and 24 h after transfection, the cells were infected with rASFV-Gluc-GFP, a recombinant ASFV expressing a report gene (luciferase) (2 MOI). After 24 h, luciferase activity assays were performed to analyze the viral replication. Data are represented as means ± SD. *p* Values were calculated with an unpaired *t* test. *B*, MA-104 cells were with the designated GFP-G3BP1 expression plasmids or empty vector, and 24 h later, infected with ASFV-HLJ/18 (ASFV-WT) (MOI of 2). After 24 h, total DNA was extracted, and the viral genome copies of ASFV were evaluated by quantitative PCR (qPCR) using TaqMan. Data are represented as means ± SD. *p* Values were calculated with an unpaired *t* test. *C*, G3BP1 knockout in MA-104 cells (MA-104-ΔG3BP1) was confirmed by Western blot. *D* and *E*, MA-104-ΔG3BP1 cells (knocking out G3BP1) were infected with rASFV-Gluc-GFP or ASFV-WT as respectively described in *A* and *B*. Data are represented as means ± SD. *p* Values were calculated with an unpaired *t* test. *F*–*H*, PAMs were transfected with scramble siRNA or siRNA targeting G3BP1 for 24 h and then noninfected or infected with ASFV for 24 h. Viral genome copies (*G*) and viral titer (*H*), respectively, detected by qPCR or HAD assay. The knockdown of G3BP1 was shown in (*F*). Data are represented as means ± SD. *p* Values were calculated with a one-way ANOVA. ASFV, African swine fever virus; G3BP1, Ras-GTPase-activating protein (SH3 domain) binding protein 1; HAD, hemadsorption; MOI, multiplicity of infection; PAM, porcine alveolar macrophage.

Cytoplasmic SGs are increasingly emerging as a critical immune signaling platform to restrict invading viral pathogens (33), whereas G3BP1 turns out to be a target of particular importance for viruses aiming to interfere with its function as an SG nucleator and regulator involved in immune responses (27). Therefore, it is reasonable to speculate that the S273R-mediated G3BP1 cleavage damages its antiviral activity. To confirm this speculation, MA-104 cells, a suitable cell line for ASFV growth, were transfected with expression plasmids encoding porcine G3BP1, G3BP1-G140A, G3BP1-N_1–140_, G3BP1-C_141–465_ or EV for 24 h, and then infected with a recombinant ASFV strain expressing a report gene (luciferase) or ASFV HLJ/18 (ASFV-WT) (34). At 24 hpi, the cells were collected to determine viral infection. As shown in Figure 6, *A* and *B*, overexpression of G3BP1 significantly inhibited ASFV replication as revealed by a viral reporter gene and viral DNA replication, whereas ASFV replication in the cells expressing G3BP-N_1–140_ or G3BP1-C_141–465_ significantly increased than in the cells expressing G3BP1-WT but had no difference with the EV control. Of note, compared with G3BP1, a stronger inhibitory effect could be detected in the cells transfected with a plasmid expressing G3BP1-G140A, which renders G3BP1 resistant to cleavage by pS273R. To exclude the impact of endogenous G3BP1, we successfully knocked out G3BP1 in MA-104 cells (designated MA-104-ΔG3BP1) (Fig. 6*C*) and then ectopically expressed G3BP1, G3BP1-G140A, G3BP1-N_1–140_, G3BP1-C_141–465_. As expected, the consistent results are shown in Figure 6, *D* and *E*. Moreover, compared with the nontargeted siRNA group, ASFV genomic DNA levels and viral titer were significantly enhanced by G3BP1 knockdown in PAMs (Fig. 6, *F*–*H*). These results suggest that the cleavage of G3BP1 by ASFV abrogates the ability to inhibit ASFV replication, whereas the mutant with the cleavage site mutation enhances the antiviral activity of G3BP1.

## Discussion

SGs are regarded as an integral part of the antiviral response of the host and therefore frequently countered by various viruses *via* sequestering or cleaving the core SG-nucleating proteins such as G3BP1. In the study, we found that G3BP1 is a new target of ASFV-encoded pS273R. ASFV pS273R interacted with and cleaved G3BP1 at the G140–F141 site. Furthermore, the G3BP1-N_1–140_ and G3BP1-C_141–465_ fragments from the G3BP1 cleavage by pS273R lost the ability to induce SG formation, which contributes to the inhibition of SG formation by ASFV infection and finally relieves the inhibitory effect of SGs on ASFV replication.

Accumulating data show that SGs exert antiviral effects in two main aspects (27). On the one hand, since SG formation is a manifestation and result of robust translation arrest, it may block viral gene expression. On the other hand, an attractive emerging view is that SGs are critical immune signaling platforms to detect and restrict invading viral pathogens. Interestingly, many viruses actively suppress SG formation to maximize replication efficiency. Our study found that ASFV did not induce SG formation like influenza A virus, Sendai virus, and Theiler’s murine encephalomyelitis virus (14) (Fig. 1). The key mechanisms that viruses use to inhibit SG formation are also becoming clearer, including viral proteins to cleave or hijack the critical SG components (12, 27, 35), to rescue translation arrest such as dephosphorylating eIF2α. Among these core components of SGs, G3BP1, as a molecular switch for SG formation, is usually countered by viral protease (poliovirus 3C protease, picornaviruses 2A, foot-and-mouth disease virus leader protease, coxsackievirus type B3 3C, and so forth) (12, 18, 36, 37, 38). According to our screening results involved in the ASFV-encoded proteins inhibiting SG formation, several ASFV proteins were identified including the cysteine protease pS273R, which showed the significant inhibitory effect (Fig. 2*A*). During ASFV infection, SG formation could be observed in the knockdown of pS273R group, compared with the ASFV-WT infected group although the percentage of ASFV-infected cells with SG formation was still low (Fig. 2, *E* and *F*). There is a possible reason that other ASFV proteins, such as pI73R and pO61R, may participate in inhibiting SG formation. In addition, our previous data showed that pS273R was associated with G3BP1 by pull-down mass spectrometry with His-S273R proteins (8). These observations prompted us to investigate the pS273R-G3BP1 interaction and the biological process regulated by this interaction. We found that pS273R interacted with and cleaved G3BP1 at the G140 site in the overexpression experiments (Figs. 3 and 4). Meanwhile, G3BP2, a G3BP1 homolog protein recruited to SGs (29), was not cleaved by pS273R (Fig. 3*D*).

During ASFV infection, an approximately 40 kDa cleavage product (designated G3BP1-C) could be detected after pS273R was expressed from 8 hpi onward (Fig. 3*F*). These data indicate that pS273R may regulate the function of G3BP1-mediated SG assembly. We found that, unlike the full-length G3BP1, G3BP1-N_1–140_ and G3BP1-C_141–465_ that mimic cleavage products generated by ASFV pS273R could not induce SG assembly. And a cleavage-resistant G3BP1 mutant (G3BP1-G140A) still triggered SG formation in the presence of pS273R (Fig. 5). Besides, several previous reports showed that viruses also target the upstream signaling of SG formation. For example, four known eIF2a kinases (PKR, PERK, HRI, and GCN2) sensed different types of environmental stresses and then are activated to intrigue eIF2α phosphorylation, ultimately converging upon SGs (14). For instance, the Kaposi’s sarcoma-associated herpresvirus ORF57 protein inhibits SG formation by blocking PKR–eIF2a activation (39). But this observation was not shown in the overexpression of pS273R. We speculated that other ASFV proteins may execute similar functions. Taken together, our results suggest that pS273R-cleaved G3BP1 inhibits SG formation.

In addition to its role as an SG nucleation factor, G3BP1 turns out to function as a regulator of immune responses, particularly in IFN response (40, 41). Two recent studies have shown that G3BP1 associates with cGAS and enhances the DNA-binding activity of cGAS to produce more IFN-β (41, 42), which depends on the integrity of G3BP1. Since the cGAS-STING pathway was inhibited in ASFV infection (31), we then investigated whether the cleavage products of G3BP1 by pS273R affect the cGAS-STING–mediated IFN pathway. We found that cleavage products of G3BP1 did neither interact with cGAS nor it promoted the binding of cGAS to ASFV DNA, as previously reported (41). Furthermore, the full-length G3BP1 enhanced the promoter of IFN-β and the downstream effective molecule (*e.g.*, interferon-stimulation gene 56) rather than its cleavage fragments (Fig. S3). These results suggest that pS273R-mediated G3BP1 cleavage impairs a G3BP1-established antiviral response. As expected, a cleavage-resistant G3BP1 (G3BP1-G140A) enhanced the antiviral activity of G3BP1 during ASFV infection (Fig. 6).

**Figure 7:**
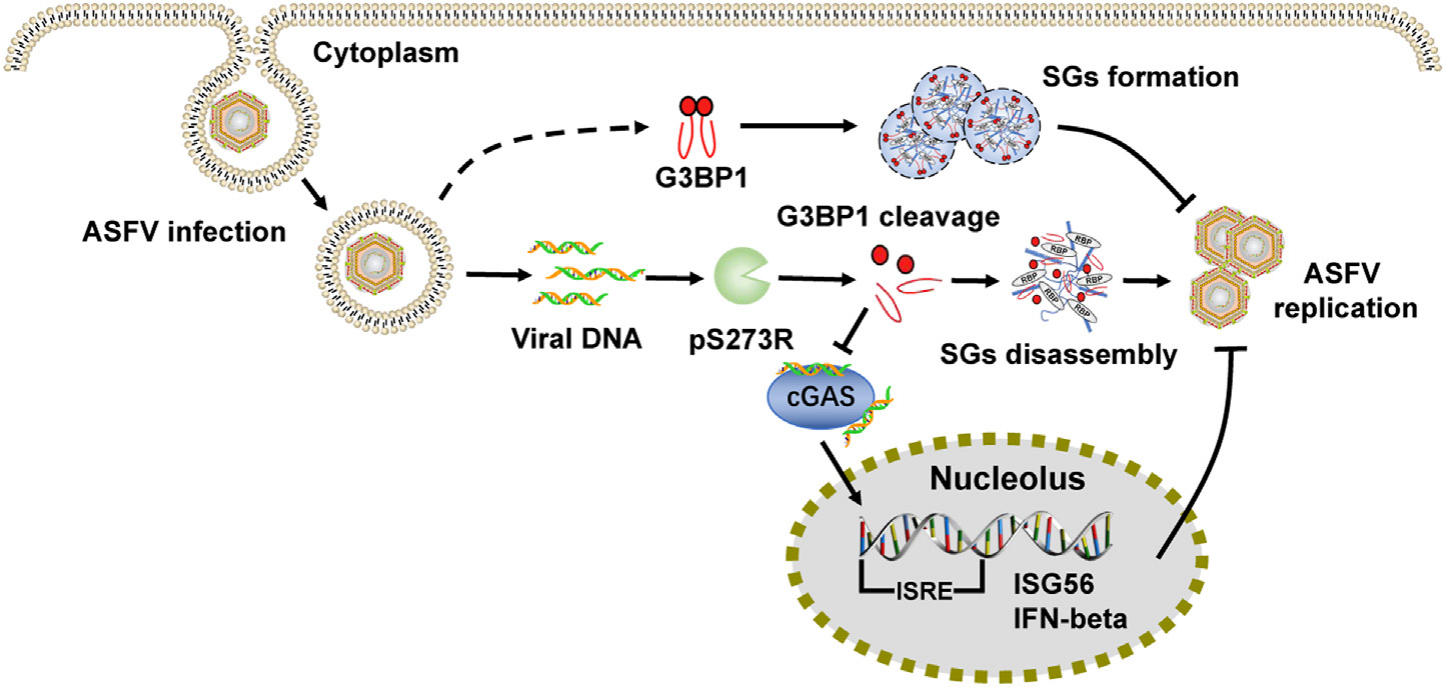
**ASFV pS273R antagonizes SG formation by cleaving G3BP1 to facilitate viral replication.** The ASFV-encoded pS273R is expressed in a late stage before viral assembly. ASFV pS273R cleaves swine G3BP1 at G140–F141 in a manner that is dependent on its protease activity. And the pS273R-cleavage products of G3BP1 lost the ability to induce SG formation and G3BP1-mediated IFN response, which may contribute to the inhibition of SG formation by ASFV infection and finally relieve the inhibitory effect of SGs on ASFV replication. ASFV, African swine fever virus; IFN, interferon; G3BP1, Ras-GTPase-activating protein (SH3 domain) binding protein 1; SG, stress granule.

In this study, we found and verified that ASFV pS273R cleaved swine G3BP1 to inhibit SG formation, which facilitated ASFV replication (Fig. 7). Interestingly, no obvious SG formation was detected in the life cycle of ASFV infection, but G3BP1 induced cleavage in the late stage of ASFV infection, indicating that other viral proteins may antagonize the process at least during the early stage. In this screening, we also found that pI73R, an early ASFV protein, also inhibited SG formation. Therefore, besides pS273R, it is necessary to investigate the inhibitory effect of ASFV proteins on SG formation, which may benefit us to understand a comprehensive biological process involved in the immune escape mechanism of ASFV.

## Experimental procedures

### Ethics statements

All experiments with ASFV HLJ/18 and rASFV-Gluc-GFP were conducted within the enhanced biosafety level 3 (P3+) facilities at the Harbin Veterinary Research Institute of the Chinese Academy of Agricultural Sciences and were approved by the Ministry of Agriculture and Rural Affairs and the China National Accreditation Service for Conformity Assessment. The protocols were approved by the Committee on the Ethics of Animal Experiments of the Harbin Veterinary Research Institute of the Chinese Academy of Agricultural Sciences and the Animal Ethics Committee of Heilongjiang Province, China (grant no.: 220303-01-GR).

### Cell lines and viruses

PAMs were isolated from specific pathogen-free piglets (without ASFV, porcine reproductive and respiratory syndrome virus, pseudorabies virus, porcine circovirus type 2, and 28 other pathogens) and cultured in RPMI1640 supplemented with 10% fetal bovine serum, 100 U/ml penicillin, and 100 mg/ml streptomycin. HEK293T cells and MA-104 cells were cultured in Dulbecco's modified Eagle's medium supplemented with 10% fetal bovine serum. All the cells were maintained at 37 °C with 5% CO_2_. The ASFV HLJ/18 strain (GenBank accession number: MK333180.1) (43) and a recombinant ASFV strain expressing a report gene (luciferase) (34) were used in the study. A hemadsorption assay was performed as described previously (44).

### Plasmids and reporters

To construct plasmids expressing GFP-tagged G3BP1, G3BP2, or double-tagged G3BP1 complementary DNA (cDNA) corresponding to the swine G3BP1, G3BP2 was amplified by standard RT–PCR using total RNA extracted from porcine alveolar macrophages as templates, and then, the cDNAs were cloned into the pEGFP-C1(pGFP) vector. The cDNAs corresponding to the site mutants of swine G3BP1 (G44A, G140A, G374A, G432A, D436A, G440A, and G448A) and the deleted mutants of G3BP1, including 1 to 140 aa, 141 to 465 aa of G3BP1, were cloned into the pGFP-C1 vector or pCAGGS-HA. The sequences of the primers used in this study are available upon request. All the constructs were validated by DNA sequencing. The 102 plasmids expressing ASFV-encoded proteins, the plasmids including FLAG-tagged S273R (FLAG-S273R), S273R-H168R, C232S, and H168R/C232S (DM), cGAS, STING, and HA-tagged S273R were preserved in our laboratory (8). The IFN-β reporter, ISG56 reporter, and thymidine kinase-Renilla reporter were obtained from Professor Hong Tang.

### Antibodies and reagents

The primary antibodies used in this study were specific for G3BP1 (Abcam; catalog no.: b181150), G3BP1 (Proteintech; catalog no.: 13057-2-AP), TIAR (Cell Signaling Technology; catalog no.: 8509S), GFP (Abcam; catalog no.: ab290), FLAG (Cell Signaling Technology; catalog no.: 14793S), HA (Cell Signaling Technology; catalog no.: 3724S), GAPDH (Proteintech; catalog no.: 10494-1-AP), ASFV p30, p72 (prepared in our laboratory), and pS273R (gifted by Professor Jianzhong Zhu). IRDye 800CW goat antimouse immunoglobulin G (IgG) (H + L) (catalog no.: 926-32210) was purchased from Sera Care, and IRDye 800CW goat anti-rabbit IgG (H + L) (catalog no.: 925-32211) was purchased from LI-COR. Alexa Flour 488 goat anti-rabbit IgG (H + L) and Alexa Flour 594 goat antimouse IgG (H + L) were all purchased from Invitrogen. The protein agarose A/G used for co-IP was purchased from Santa Cruz Biotechnology (catalog no.: 20397). Sodium arsenate (Sigma–Aldrich; catalog no.: S7400) was dissolved in water at the storage concentration of 500 mM.

### Generation of MA-104-G3BP1 and HEK293T-G3BP1 knockout cell line

As previously described, CRISPR–Cas9 genomic editing for gene deletion was used (45). MA104-ΔG3BP1 and HEK293T-ΔG3BP1 cell lines were constructed using the CRISPR–Cas9 method. To create ΔG3BP1 cells, one CRISPR guide RNA (single guide RNA [sgRNA]) sequence targeting the G3BP1 locus in the genome was chosen based on the specificity scores (http://crispr.mit.edu/). The sgRNA sequence was used as follows: G3BP1 sgRNA, 5′-GGAGAAGCCTAGTCCCCTGC-3′.

### Co-IP and Western blot analysis

Co-IP and Western blot analysis were performed as described previously (46). HEK293T cells transfected with the indicated plasmids for 24 h were lysed with cell lysis buffer (50 mM Tris–HCl, pH 7.4, 150 mM NaCl, 5 mM MgCl_2_, 1 mM EDTA, 1% Triton X-100, and 10% glycerol) containing 1 mM PMSF (Beyotime) and 1× protease inhibitor mixture (MedChemExpress). The cell lysates were incubated with anti-FLAG (M2) beads (Sigma; catalog no.: A2220-5ML) overnight at 4 °C on a roller. The immunoprecipitants were subjected to electrophoresis. In addition, to identify the interactions between endogenous proteins, PAMs were noninfected or infected with ASFV (1 MOI) for 36 h. The cell lysates then were incubated with an anti-S273R polyclonal antibody or IgG for 8 h at 4 ˚C, and S273R complexes were captured using protein A + G-Sepharose. For Western blotting analysis, equal amounts of cell lysates and immunoprecipitants were resolved by 12% SDS-PAGE and then transferred to polyvinylidene difluoride membranes (catalog no.: ISEQ00010; Merck-Millipore). After incubation with primary and secondary antibodies as indicated, the membranes were visualized by an Odyssey two-color infrared fluorescence imaging system (LI-COR).

### Fluorescence microscopy

The cells were transfected with the indicated plasmids or infected with ASFV. And then fixed for 10 min in 4% paraformaldehyde in 1× PBS (pH 7.4). The fixed cells were permeabilized for 15 min with 0.3% Triton X-100 in 1× PBS and then blocked in 1× PBS with 5% bovine serum albumin for 30 min. The cells were incubated with the indicated primary antibodies and then stained with Alexa Fluor 488-labeled goat anti-rabbit IgG and Alexa Fluor 594-labeled goat antimouse IgG. The SG formation stained with G3BP1 or TIAR antibodies and the subcellular colocalization of pS273R and G3BP1 were visualized using a Zeiss LSM-800 laser scanning fluorescence microscope (Carl Zeiss AG) under a 63× oil objective.

### Quantification of SG-positive cells

According to a previous report (47), to determine the number of SG-positive cells, at least 15 fields 63× images were captured per experiment. Cells displaying punctate immunofluorescent foci of G3BP-1 were considered as SG positive. The percentage of SG-positive cells was determined in the infected cells or cells expressing indicated plasmids in at least 15 random fields.

### Quantitative PCR

To test the full-length G3BP1, its cleavage fragments and G3BP1 mutant (G3BP1-G140A) for ASFV replication, MA-104 cells or MA-104-△G3BP1 cells were transfected with indicated plasmids (0.5 μg per well) for 24 h, followed by ASFV infection with 2 MOI. At 24 hpi, the cells were harvested, and ASFV genomic DNA was extracted from cells using a Qiagen DNA Mini Kit (Qiagen). Quantitative PCR was carried out on a QuantStudio5 system (Applied Biosystems) according to the OIE-recommended procedure. All the quantitative PCR primers are listed in Table S1.

### siRNA assay

siRNAs that target swine G3BP1, ASFV S273R, or nontargeting siRNAs (siNC) were chemically synthesized (GenePharma, Incorporated). In brief, PAMs were transfected with the corresponding siRNA using Lipofectamine RNAiMAX Transfection Reagent from Invitrogen according to the manufacturer’s instructions. At 24 h post-transfection, the cells were infected with ASFV HLJ/18 at 1 MOI for 24 h. The cells were collected, and the viral replication was analyzed by quantitative RT–PCR and hemadsorption assay described as previously reported (44). The siRNAs used in this study are listed in Table S1.

### *In vitro* DNA-binding assay

For the biotin–DNA-binding experiment, the assay was performed according to a previous report (48). Briefly, a total of 20 μg ASFV genome DNA was labeled with 20 μg photobiotin under strong light (450 W, 220 V), and the biotin–DNAs were incubated with an equal amount of the HEK293T cell lysates overexpressing FLAG-cGAS together with GFP-G3BP1 or GFP-G3BP1-N_1–140_, GFP-G3BP1-C_141–465_, or GFP. Then, the biotinylated DNA–protein complexes were precipitated with streptavidin agarose resin (Invitrogen) and analyzed by Western blotting with specific antibodies for FLAG, GFP, and GAPDH.

### Luciferase reporter assay

According to the manufacturer’s instructions, luciferase activities were measured with a Dual-Luciferase Reporter Assay System (Promega), and the data were normalized to the transfection efficiency by dividing the firefly luciferase activity by the Renilla luciferase activity.

### Statistics

All the statistical analyses were performed using GraphPad Prism 8 software (GraphPad Software, Inc). Data are presented as the mean ± SD, and *p* values were calculated with a Student’s *t* test or a one-way ANOVA.

## Data availability

All relevant data are within the article and its supporting information files.

## Supporting information

This article contains supporting information.

## Conflict of interest

The authors declare that they have no conflicts of interest with the contents of this article. The funding source was not involved in the analysis or the preparation of this report. The corresponding authors had full access to all the data and accepted the final responsibility for the decision to submit this article for publication.
