# Supplementary material for: African swine fever virus pS273R antagonizes stress granule formation by cleaving the nucleating protein G3BP1 to facilitate viral replication
Source: J Biol Chem. 2023 May 19;299(7):104844. doi: 10.1016/j.jbc.2023.104844 (PMC10404608; doi:10.1016/j.jbc.2023.104844)
Supplement: Table S1 [file mmc1.docx]

**Table S1. The sequences of the qPCR primers and siRNA used in the study**

| Purpose | name | Sequence (5′-3′) |
| --- | --- | --- |
| siRNA sequences | siRNA-G3BP1-1 | GGUGGUGCCUGAUGAUUCUTT |
|  |  | AGAAUCAUCAGGCACCACCTT |
|  | siRNA-G3BP1-2 | GGACAAGUUAGAGCUUAAATT |
|  |  | UUUAAGCUCUAACUUGUCCTT |
|  | siRNA-S273R-1 | GCGCAGAGCAUCUUACAAATT |
|  |  | UUUGUAAGAUGCUCUGCGCTT |
|  | siRNA-S273R-2 | CCGACGAAGACAUGUAUAATT |
|  |  | UUAUACAUGUCUUCGUCGGTT |
|  | siRNA-NC | UUCUCCGAACGUGUCACGUTT |
|  |  | ACGUGACACGUUCGGAGAATT |
| qPCR primers | *ASFV-p72* | CTGCTCATGGTATCAATCTTATCGA |
|  |  | GATACCACAAGATCAGCCGT |
|  |  | FAM-CCACGGGAGGAATACCAACCCAGTG-TAMRA |
