# Supplementary material for: African swine fever virus pS273R antagonizes stress granule formation by cleaving the nucleating protein G3BP1 to facilitate viral replication
Source: J Biol Chem. 2023 May 19;299(7):104844. doi: 10.1016/j.jbc.2023.104844 (PMC10404608; doi:10.1016/j.jbc.2023.104844)
Supplement: Supporting information [file mmc2.docx]

**African swine fever virus pS273R antagonizes stress granule formation by cleaving the nucleating protein G3BP1 to facilitate viral replication**

Tingting Li ^1, 2,a^ , Xuewen Li^1, 2,3,a^ , Xiao Wang^1, 2^, Xin Chen^1, 2^, Gaihong Zhao^1, 2^, Chuanxia Liu^1, 2^, Miaofei Bao^1, 2^, Jie Song^1, 2^, Jiangnan Li^1, 2^, Li Huang^1, 2^, Jun Rong^3^, Kegong Tian^4^, Junhua Deng^5^ , Jianzhong Zhu^6^, Xuehui Cai^1^, Zhigao Bu^1^, Jun Zheng^1, 2, *^, Changjiang Weng^1, 2, *^

^1^ Division of Fundamental Immunology, State Key Laboratory for Animal Disease Control and Prevention, Harbin Veterinary Research Institute of Chinese Academy of Agricultural Sciences, Harbin 150069, China.

^2^ Heilongjiang Provincial Key Laboratory of Veterinary Immunology, Harbin 150069, China.

^3^ College of Life Sciences, Yangtze University, Jingzhou 434025, China.

^4^National Research Center for Veterinary Medicine, Luoyang 471003, China.

^5^ Luoyang Putai Biotechnology Co., Ltd, Luoyang 471003, China.

^6^College of Veterinary Medicine, Yangzhou University, Yangzhou 225009, China.

^a^ Tingting Li and Xuewen Li contributed equally to this work.

^*^Corresponding authors: Changjiang Weng and Jun Zheng.

Division of Fundamental Immunology, Harbin Veterinary Research Institute, Chinese Academy of Agricultural Sciences (CAAS), Harbin 150069, China

Email: [wengchangjiang@caas.cn](mailto:Wengcj@caas.cn) and zhengjun01@[caas.cn](mailto:Wengcj@caas.cn)

## Supplementary Figures


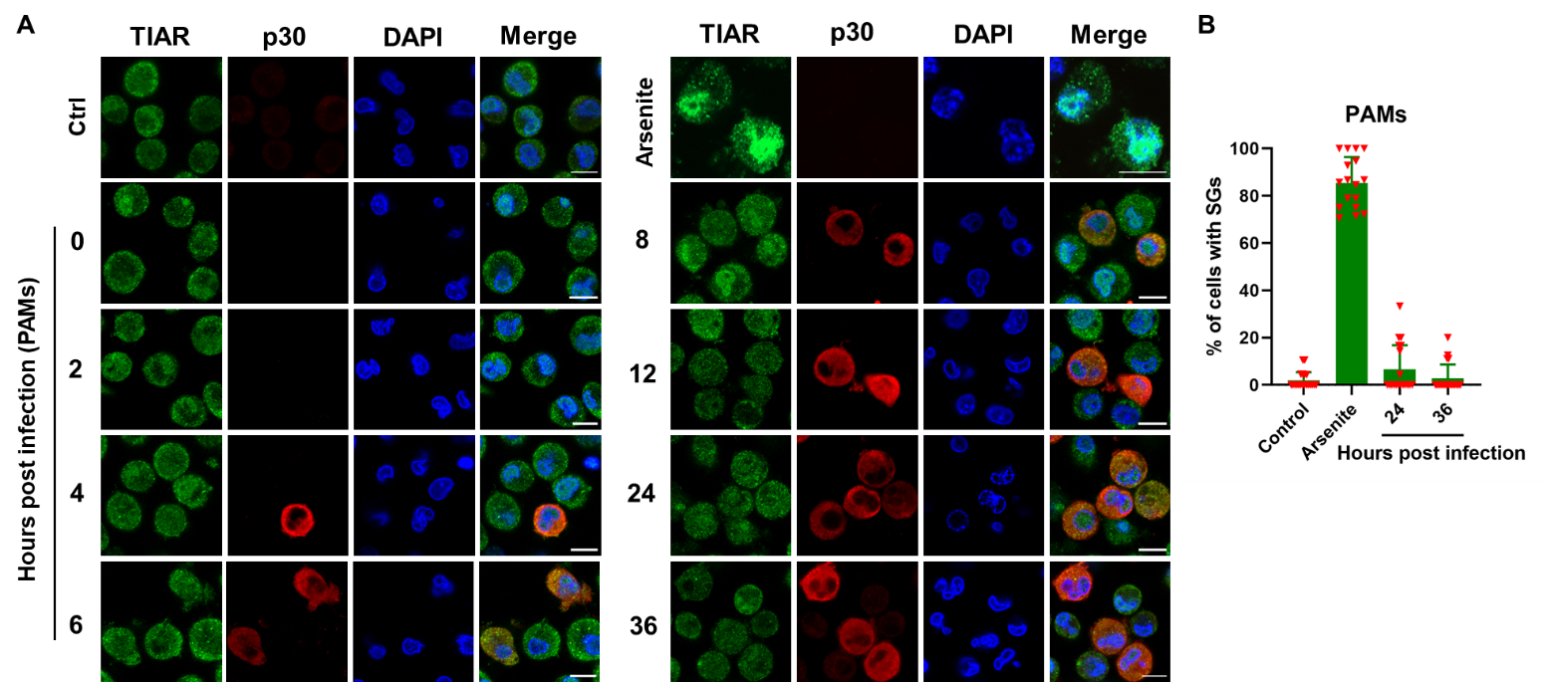


## Figure S1. ASFV infection inhibits the formation of SGs in PAMs.

(A) PAMs were infected with ASFV-WT (MOI of 1), fixed at indicated time points. And cells were treated with arsenite (0.5 μM) for 1h as a positive control. The cells were fixed and stained with either rabbit monoclonal specific antibodies for TIAR (green) or mouse polyclonal specific antibodies for p30 (red). Nuclei were stained with DAPI (blue). The cells were analyzed by confocal microscopy. Scale bar =10 μM. (B) The percentage of SGs positive cells to infected cells at an indicated time point, which was calculated in 15 random fields, presented as mean ± SD. *P*-values were calculated with an unpaired *t*-test.


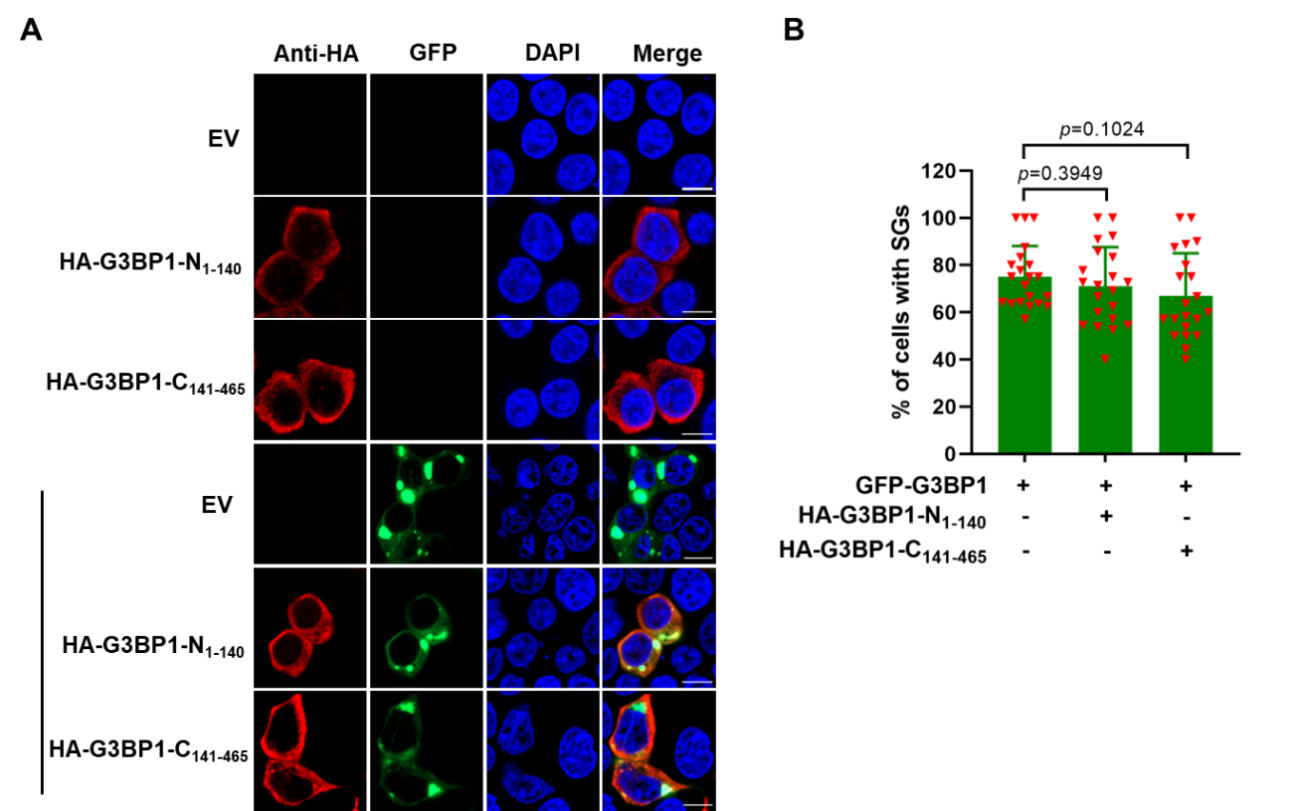


## Figure S2. The pS273R-cleaved products of G3BP1 did not interfere with its ability to promote SGs formation.

(**A**) HEK293T cells were transfected with a GFP-G3BP1 expression plasmid respectively together with HA-G3BP1-N_1-140_, HA-G3BP1-C_141-465_ or empty vector (EV) expression plasmids respectively. The cells were then fixed and stained with HA-tagged antibodies for HA-G3BP1-N_1-140_ and HA-G3BP1-C_141-465_ (red). Nuclei were stained with DAPI (blue). The cells were analyzed by confocal microscopy. Scale bar =10 μM. (**B**) The percentage of cells containing SGs out of cells expressing GFP-G3BP1 alone or together with HA-G3BP1-N_1-140_ or HA-G3BP1-C_141-465_ was calculated in 20 random fields in (A). Data are represented as means ± SD. *P*-values were calculated with an unpaired *t*-test.


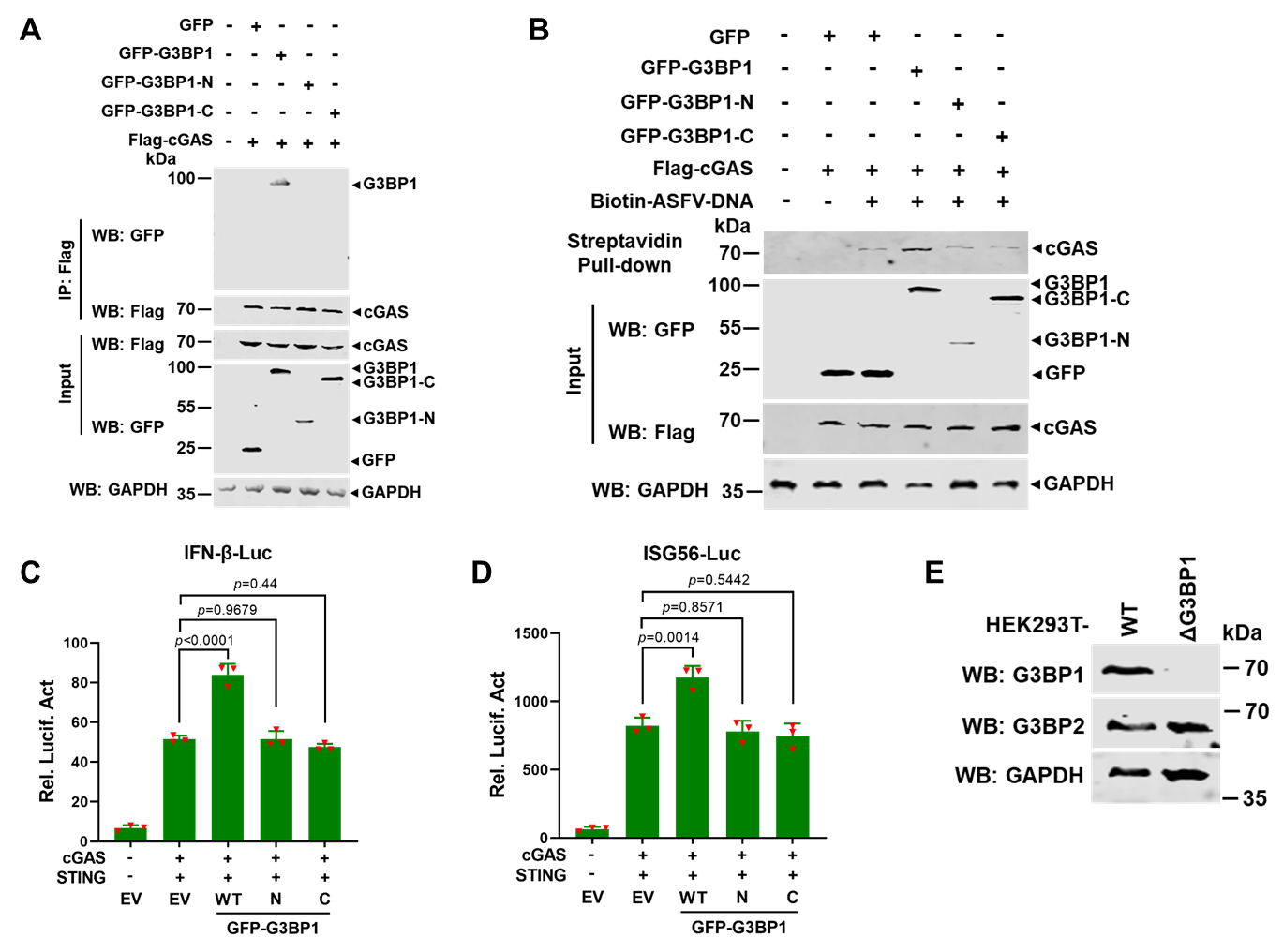


## Figure S3. The G3BP1 fragments cleaved by pS273R are unable to induce type I interferon production.

(A) The G3BP1-cGAS interaction was analyzed by immunoprecipitation with anti-Flag beads in HEK293T cells expressing Flag-tagged cGAS and GFP-tagged the full-length G3BP1 or the pS273R-cleaved fragments of G3BP1 (GFP-G3BP1-N and G3BP1-C). (**B**) Analysis of the effect of G3BP1 on ASFV DNA-bound cGAS by western blotting. ASFV DNA was labeled with biotin and then incubated with the cell lysis from cGAS-deficient HEK293T cell ectopically expressing Flag-cGAS and GFP-G3BP1 or pS273R-cleaved products (GFP-G3BP1-N and G3BP1-C), followed by a streptavidin pull-down to precipitate the DNA-bound Flag-cGAS. (C, D) HEK293T-ΔG3BP1 cells (knockout G3BP1) were transfected with an IFN-β (C) or an ISG56 (D) luciferase (Luc) reporter and a Renilla-TK reporter and plasmids expressing HA-cGAS and HA-STING, together with a plasmid expressing GFP-G3BP1 or GFP-G3BP1-N1-140 and G3BP1-C141-465 for 24 h. The cells were collected to detect the Luc activity. Data are represented as means ± SD. *P*-values were calculated with a one-way ANOVA. (**E**) G3BP1 knockout in HEK293T cells was confirmed by Western blotting.


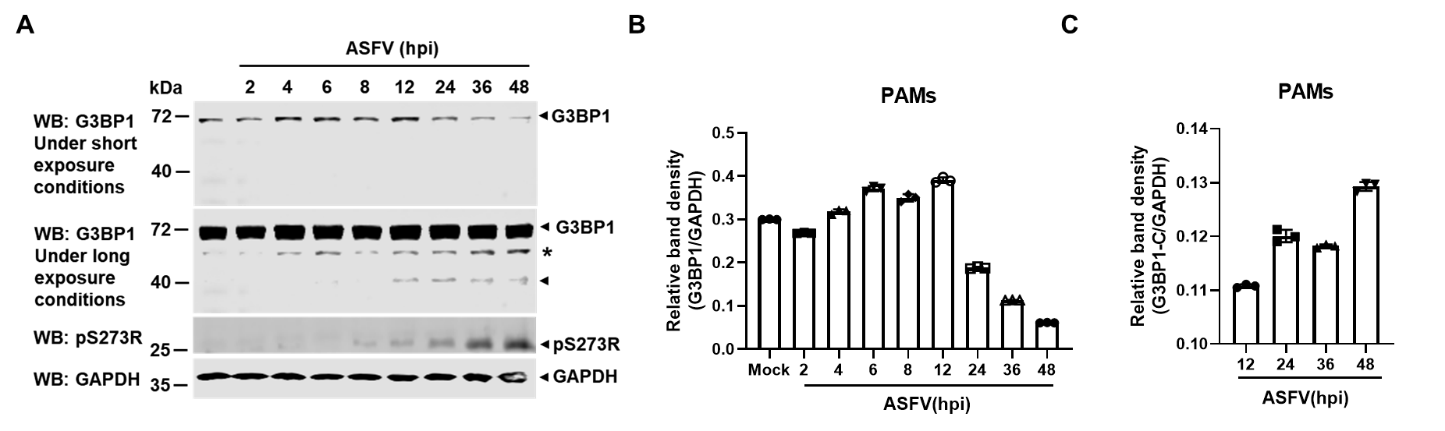


## Figure S4. G3BP1 is cleaved during ASFV infection.

**(A)** PAMs were mock infected or infected with ASFV at MOI of 1. At the indicated time points, the cell lysates were analyzed by Western blot with antibodies against G3BP1, pS273R, and GAPDH. *: indicated the non-specific bands. **(B-C)** Quantitation of G3BP1/GAPDH ratio(B) or G3BP1-C/GAPDH ratio (C) from ImageJ analysis in (A).

## Table S1. The qPCR primers and siRNAs sequences were used in this study.
